# Multi-culture label-free quantitative cell migration sensing with single-cell precision

**DOI:** 10.1364/BOE.541010

**Published:** 2024-12-20

**Authors:** Piotr Arcab, Mikołaj Rogalski, Marcin Marzejon, Piotr Rogujski, Luiza Stanaszek, Maciej Trusiak

**Affiliations:** 1 Warsaw University of Technology, Institute of Micromechanics and Photonics, 8 Sw. A. Boboli St., 02-525 Warsaw, Poland; 2 NeuroRepair Department, Mossakowski Medical Research Institute, Polish Academy of Sciences, 02-106, Warsaw, Poland; 3 piotr.arcab.dokt@pw.edu.pl; 4 maciej.trusiak@pw.edu.pl

## Abstract

A fair comparison of multiple live cell cultures requires examining them under identical environmental conditions, which can only be done accurately if all cells are prepared simultaneously and studied at the same time and place. This contribution introduces a multiplexed lensless digital holographic microscopy system (MLS), enabling synchronous, label-free, quantitative observation of multiple live cell cultures with single-cell precision. The innovation of this setup lies in its ability to robustly compare the behaviour, i.e., migratory pathways, of cells cultured or contained in different ways (with varied stimuli applied), making it a valuable tool for dynamic biomedical diagnostics on a cellular level. The system's design allows for potential expansion to accommodate as many samples as needed, thus broadening its application scope in future quantitative diagnostics on global multi-culture cellular behaviours via their localized single-cell spatiotemporal optical signatures. We believe that our method has the potential to empower reliable live cell multi-culture comparisons through simultaneous quantitative imaging, enhancing label-free investigations into cell cultures and the effects of biochemical or physical stimuli over large areas, and unlocking novel mechanistic understandings through high-throughput time-lapse observations.

## 1. Introduction

The study of living cells is a fundamental aspect of biology and biomedicine. The examination of cell cultures’ spatiotemporal characteristics over time is important as they reveal global and statistically relevant information about ensemble mobility and interactions. Especially, cell migration plays a crucial role in physiological and pathological biological processes such as wound healing [1] or cancer metastasis [2]. Different cells within the whole organism migrate in their environment/niche to perform their natural functions. There are many different examples of such behavior starting from the migration of lymphocytes toward the pathogen, or migration of oligodendrocytes to produce myelin around the axons, or stem cells migrating to their place of destination where upon reaching they start to differentiate and perform their functions. The latter type of migration is pivotal in stem cell-based therapies [3], where cells after transplantation need to migrate toward the injury site to repair the damaged area or help to establish homeostasis to prevent further impairment.

Given the critical role of cell migration in various physiological and pathological processes, researchers have developed several methods to study this phenomenon in vitro [4,5]. One commonly used method to study cell migration is the “scratch assay” [6]. This method involves: (1) creating a scratch in a monolayer cell culture using a pipette tip or another standardized tool, (2) continuing the cell culture, and finally (3) imaging and measuring the degree of scratch closure over time. Ideally, the rate of scratch closure should correlate with the rate of cell migration in the culture. However, altered cell proliferation and the degree of mechanical damage can hinder the reliable analysis of results and the reproducibility of the invasive experiment [6]. Moreover, making a scratch on a culture dish coated with extracellular matrix proteins can damage the protein layer, disrupting the migration of the cells being studied [7,8]. These limitations highlight the need for noninvasive, label-free methods to monitor individual cell dynamics without damaging the cell culture.

Additional problems arise when quantitatively comparing different cell cultures sequentially, due to variations in environmental conditions over time [9]. Maintaining constant temperature [10], pressure [11], and humidity [12] during separate measurements is challenging and can be very expensive. Small changes in any of these parameters over time can have unknown effects on cell cultures, resulting in overall incomparable results of sequentially analyzed samples. To address this challenge, it is essential to compare different cell cultures simultaneously and within an identical setup. Moreover, it is to be noted that a parallel and fair comparison of various cell cultures (e.g., stimulated with varying dose of tested drug) prepared in the same growth process is vital, as only then we can bypass the need to prepare multi-cultures in a sequential, thus fluctuation-dependent and potentially erroneously heterogenous, way.

Presently, microscopes can picture various cell cultures places in multiple wells, but it is often related to a loss of focus during the well-to-well shift [13], especially when the cells are dispersed and highly motile. Additionally, the temporal resolution can be compromised due to the time required for shifting, highlighting the need to develop microscopic methods with higher throughput. Theoretically, these limitations may be bypassed by simultaneous imaging within multiple identical microscope setups, but this can significantly increase the costs and complexity of measurement (also personnel-related), making it an impractical solution. Standard brightfield microscopes [14,15] exhibit two additional features not favorable in terms of full-culture cell migration sensing: (1) they deploy lens-based objectives with important trade-off in terms of reducing the field of view (FOV) when increasing the resolution [16] resulting in only a small fraction of entire cell culture being studied; (2) they are sensitive to light absorption while most live cells are nearly transparent [17] and thus very difficult to be efficiently observed.

Quantitative phase imaging (QPI) [18,19] techniques allow for higher contrast imaging of translucent samples and provide quantitative information about their optical thickness. Holographic QPI methods [20,21] generate a hologram through coherent interference of object and reference beams. Among various holographic configurations, digital in-line holographic microscopy (DIHM) [22–24] is notable for its hardware simplicity. The DIHM, inspired by the seminal Gabor concept [25], requires the sample to be weakly scattering. This means that some of the light passes through the sample scattered, while the majority is unscattered (ballistic). The resulting common-path defocused hologram is recorded by the camera and needs numerical backpropagation to reconstruct sharp phase/amplitude image. Deployed microscope objectives impose similar FOV restrictions as in regular brightfield microscopes, however.

To address the challenge of large FOV imaging, lensless digital holographic microscopy (LDHM) was introduced [26–29] as a variation of DIHM that operates without lenses, making it also a cost-effective solution. The primary advantages of LDHM are its simplicity and large FOV. In its most straightforward form, it consists of only three elements: a light source, a sample, and a sensor, all positioned on the same optical axis. The development of LDHM has accelerated with advances in sensor technology. The trend towards smaller pixel size has significantly improved the resolution of LDHM. Nonetheless, resolution in LDHM is limited by several factors, including sensor size, pixel size, numerical apertures (of illumination and detection), spatial and temporal coherence of the light source, and twin-image effect [28,30]. Popular LDHM setups place the sample close to the camera, achieving reasonable resolution close to the pixel size with a magnification around unity [27,28,31,32]. This configuration results in maximized FOV to approximately the size of the sensor. The combination of reasonable resolution (1-2 µm) and a large FOV (easily surpassing 100 mm^2^) [33] makes LDHM an ideal tool for examining entire cell cultures [34,35] or bacteria colonies [36] with single-cell/bacteria sensitivity. A single measurement can straightforwardly capture hundreds-to-thousands of cells, making it an ideal tool for full-culture migration measurements. Additionally, LDHM allows for the observation of cells in conditions close to their natural environment. Most cells grow in complete darkness, a condition that is generally difficult to replicate in microscopy. LDHM enables label-free imaging in low-photon conditions without a loss in resolution, making it suitable for examining live cell cultures [37].

In this contribution we propose a novel method for fair comparison of living cellular samples using a multiplexed LDHM system (Multiplexed Lensless System - MLS). The novel MLS rigidly combines identical LDHM sub-setups (with a shared single light source), allowing for the simultaneous examination of label-free cell cultures. We deployed novel MLS, boosted by a new algorithmic path for robust cell space-time labeling and tracking, for time-lapse examination of living cell migration activity. As proof-of-concept verification of the MLS, we investigated the influence of a drug (here: neuregulin-1) onto a cell culture (here: mouse glial restricted progenitors mGRPs) migration activity – one of a many potential applications of the proposed system for simultaneous multi-culture label-free dynamic examination.

The rest of the manuscript is divided into four sections. The first one describes the hardware of the proposed experimental setup in detail. Second section explains the proposed method for quantitative migration sensing. The following one presents the achieved imaging and analysis results, and the last section offers discussion and conclusions.

## 2. MLS experimental setup description

The MLS was developed based on multiplexing a single LDHM system [37,38]. The setup was designed to maintain consistent distances between components throughout the entire imaging process. Furthermore, all MLS sub-units are built from the identical off-the-shelf elements, ensuring uniform FOV, magnification, and resolution. In the presented experiment we designed a 3-channel MLS, however it can be easily upgraded to N-channel MLS. The main cost of system expansion is associated with additional cameras as only one laser and a single linear stage employed. This makes our solution cost-effective, compared to classical microscopy systems. The limit on the number of LDHMs in MLS method is defined by setup construction itself (length of rails, camera holder dimensions, the load capacity of the linear stage, etc.).

**Fig. 1.**
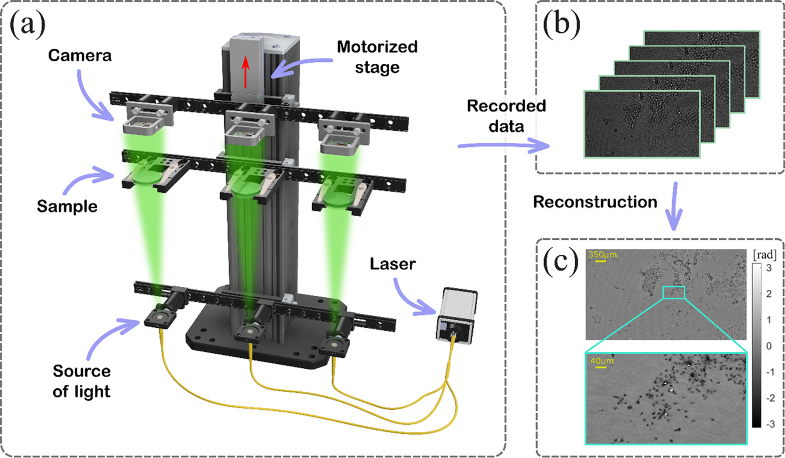
The MLS working principle: (a) 3D scheme of 3-channel MLS setup containing single source of light and three identical LDHMs, (b) five holograms recorded at different defocus distances and used for a single phase reconstruction, (c) exemplifying large FOV phase reconstruction with enlarged insert.

Figure 1 presents the proposed MLS architecture along with an exemplifying set of holograms captured by one of the sensors in the system, as well as the corresponding reconstruction. To ensure uniform illumination, we utilized a monochromatic continuous-wave laser (CNI Lasers MGL-FN-561-20 mW, λ = 561 nm, Δλ FWHM = 47 pm), coupled into an optical fiber coupler whose ends were treated as quasi-point sources of light. The use of a single laser source allows one to avoid differences in the recorded holograms caused by channel-to-channel laser output power level fluctuations. All three fiber ends were mounted on the same rail to provide parallel illumination, with carefully chosen distances between them to prevent parasitic interference. These distances were determined by the numerical aperture (NA) of the fibers (0.12-0.14) and were approximately 10 cm long in the proposed setup. To mitigate the influence of parasitic light coming from adjacent channels, light separators were inserted between individual LDHMs. They were not marked in Fig. 1 to maintain a clear presentation of the concept.

On a separate rail, three identical sample holders (Thorlabs MAX3SLH) were attached. This rail was positioned to ensure that the sample plane were near the camera plane, with a distance of approximately 15 mm due to the dimensions of the petri dish on which the cells were placed (14 mm height). Monochrome cameras (IDS U3-38J2XLE-M Rev.1.1) were mounted on the third rail, motorized by a linear translational stage (Thorlabs KMTS25E/M). The direction of the stage movement is marked with a red arrow in the Fig. 1. For one timestamp, we acquired five frames at different distances between the sample plane and the camera plane (from 15 mm to 19 mm with 1 mm step) to enhance the signal-to-noise level by suppressing the twin-image contamination in the reconstructed sample’s image. The distance between the light source plane and the camera plane was approximately 15 cm. Due to the geometric configuration, the optical magnification was approximately equal to one. To further reduce the distance between the camera and the sample, one could consider positioning the camera beneath the petri dish. However, as the system was primarily designed for cell migration sensing, where cell cultures require liquid nutrient media, placing the camera below petri dish could cause media evaporation due to the camera heat.

## 3. New algorithmic path for robust spatiotemporal cell segmentation

The object being measured in LDHM can be described as a complex optical field characterized by both amplitude and phase components. Illuminating the sample in LDHM results in propagating this optical field in free space, along with common-path unscattered reference beam, to the camera plane [39–41]. The camera enables one to record only the amplitude part of the optical complex field, which in LDHM is called an in-line hologram. A hologram is a diffractive interference pattern resulting from the interaction between light scattered by the sample and the unscattered part of the illuminating beam. Recorded hologram can be then numerically backpropagated to the sample plane, retrieving in-focus amplitude and phase components of the sample. However, as the camera detects only the amplitude and lacks phase information, the reconstruction of the hologram at the object plane is spoiled by a so-called twin-image effect. This fundamental issue impacts both resolution and measurement quality of the investigated object’s image. This pivotal problem may be addressed by either hardware modifications of the measurement setup [33,42–44] or by software twin-image suppressing via iterative algorithms [45–47].

One of the most widely used algorithms for minimizing the twin-image error is the Gerchberg-Saxton (GS) algorithm [48,49]. This algorithm necessitates at least two holograms that differ from each other in propagation distance [50,51] or wavelength [52–55]. Here, each reconstruction was calculated based on 5 frames acquired at propagation distances differing by one millimeter from the previous frame. For results presented in the next section, a set of five frames was captured every 5 minutes for 68.5 hours. All reconstructed series of holograms are presented in the Supplementary Visualization 1, Visualization 2 and Visualization 3. The requirement of capturing five frames for each measurement limits the temporal resolution of the proposed system to over five times the single frame acquisition time (see Appendix). Despite this limitation, the system was still adequate for the mGRPs cells measured in this article, which at their fastest move at a speed of 1 pixel per minute. If higher temporal resolution is required, alternative methods can be easily employed. For instance, a single-frame setup with a neural network [37] or an RGB illumination and acquisition setup [55] may be used, allowing data acquisition at the camera's framerate.

**Fig. 2.**
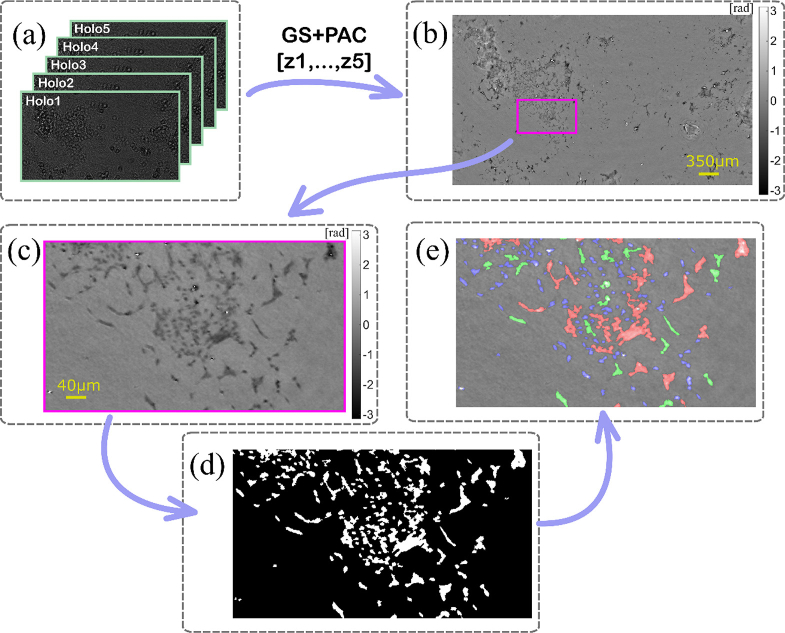
The MLS processing path: (a) set of five holograms with different defocus distances recorded by camera, (b) phase reconstruction using 5-frame GS + PAC method, (c) enlarged area marked with pink rectangle, (d) binarized cell image, (e) size-labeled cell image (blue – individual, green – small clusters, red – large clusters).

Figure 2 presents a scheme of the proposed procedure for processing a single cell culture measurement consisting in efficient constrained phase retrieval for each timestamp and further robust spatiotemporal segmentation and tracking of cells. Its feasibility is boosted by high signal-to-noise ratio and signal-to-background ratio provided by efficient constrained phase retrieval, with complex field filtering support, originally proposed for multi-wavelength data [55], employed here for the first time in multi-height lensless regime. Every individual reconstruction was generated from a set of five recorded holograms with varying propagation distances (Fig. 2(a)). These holograms were captured in a lensless system where slight changes in geometric distances influenced varying magnification. To address this, the holograms were firstly scaled to mitigate the varying magnification's impact (see Appendix). Next, the defocus distances for each hologram were calculated using the DarkFocus autofocusing algorithm [56]. After that, the phase at the object plane was reconstructed with GS algorithm supported with additional positive absorption constraint (PAC – assuming that cells can only absorb light or be transparent, and do not produce light), which helps to improve reconstruction signal-to-noise and signal-to-background ratios. Further details about the novel GS + PAC algorithm can be found in the Appendix. An exemplifying phase reconstruction and an enlarged ROI are depicted in Figs. 2(b) and 2(c), respectively, to visually corroborate the algorithm’s efficiency. Subsequently, after the sample’s optical field reconstruction, the phase was binarized (Fig. 2(d)) using a manually determined threshold. It is noteworthy that the selected threshold remained constant throughout the experiment and across all three datasets coming from different channels. While this basic segmentation approach is sufficient due to the high signal-to-noise ratio, more challenging scenarios may require advanced techniques [57].

Following the binarization process, cells were classified into three groups based on the size of each binarized and segmented object in the image. Single cells, with an area of approximately 23 µm^2^, were categorized into the first group called, which included the majority of the observed cells. The second group consisted of small clusters of cells that could not be separated during the binarization process and had an area between 23 and 46 µm^2^. The third group included large clusters of cells occupying an area greater than 46 µm^2^. Details can be found in the Appendix. Exemplifying results of segmentation are presented in Fig. 2(e), where individual cells, small clusters, and large clusters are marked in blue, green and red, respectively.

## 4. Cell cultures preparation and migration sensing results

To experimentally validate the proposed MLS, we selected an exemplifying case of studying the potential influence of neuregulin-1 on the increase of migration speed of the mGRPs cells. Neuregulin-1 was already reported as a very interesting protein for promotion of proliferation and migration of multiple cell types [58–60], and mGRPs were chosen as their increased migration activity could potentially lead to augmented therapeutic effects in neuro-damages [3,61]. Cell growth was carried out under standard culture conditions in culture vessels coated with poly-L-lysine/laminin. mGRPs after thawing were cultured in the dedicated GRP medium on the T25 culture dishes (Falcon) until confluent. Upon seeding, recombinant neuregulin-1 (Cat. No. 9875-NR-050, R&D Systems, Minneapolis, MN, USA) was added in two different concentrations - 10 and 50 ng/ml of medium (further described as NRG10 and NRG50 respectively). The medium was changed every 2-3 days during the whole culture. Before the recording (approximately 7 days) cells were passaged into the glass-bottom dishes (Cellvis) at a density of 6 × 10^4^. Reconstruction of every sample is presented in Visualization 1, Visualization 2 and Visualization 3, presenting control cell culture, cell culture with NRG10 and NRG50, respectively. To distinct changes caused by the different concentration of neuregulin-1, every frame was binarized and segmented. With the segmented frames and knowledge about the positions of cells, it became feasible to track every single cell frame by frame (see Appendix).

**Fig. 3.**
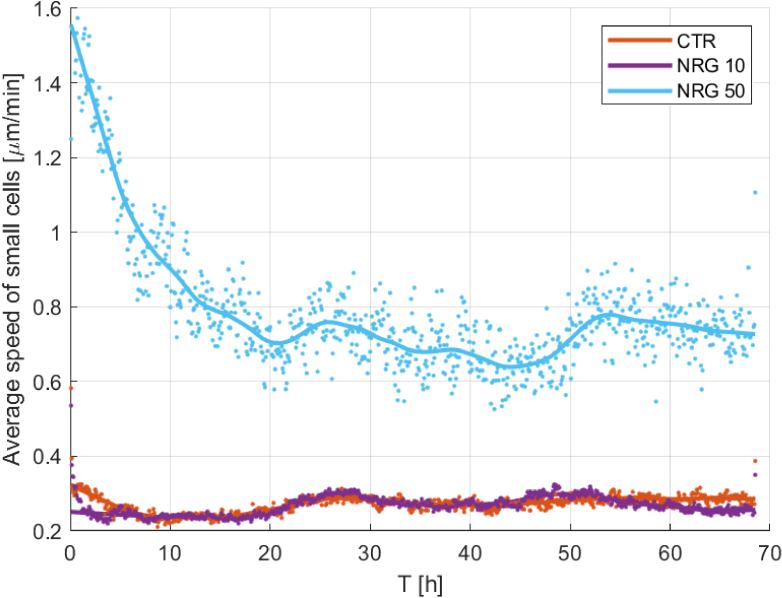
Average speed of individual cells. Cells treated with NRG50 are depicted in blue, cells treated with NRG10 in purple, and the control cell culture (CTR) in orange.

Figure 3 compares the calculated speed of living cells contained in three different samples examined simultaneously under the same external conditions. Each dot denotes the median speed of the individual cells at a given frame, with curves added to facilitate a clearer comparison of the changing speed over time. Speed of each cell was calculated by dividing the distance it travelled over the last 50 frames (previous 250 min) by the time taken to record these frames. Dot-plots presented in Fig. 3 were finally smoothed to obtain approximative curves. When contrasting the control culture with the NRG10 culture, the results are comparable with each other, as the curves oscillate around the same level of speed. However, when comparing both the control and NRG10 cultures to the NRG50 group, a distinct influence on the speed of individual cells becomes evident. Individual NRG50 cells exhibit a speed approximately 2.5 times faster than those in the NRG10 and control groups. This serves as a proof-of-concept of the proposed MLS sensing device and may lead to new biological findings in the future through quantitative spatiotemporal analysis of live cell cultures through their optonumerical signatures and heir fluctuations in time.

It is to be highlighted that the proposed system is not limited to selected exemplifying migration analysis of mGRPs cells. Setup's versatility enables simultaneous in vitro high-throughput evaluation of multiple different living cell cultures, providing meaningful insights into cells’ response to varying stimulation (e.g., electromagnetical, mechanical, chemical etc.) in real-time, regarding their migration but also potentially processes like proliferation, clusters formation and apoptosis. It is to be noted that MLS enables parallel and fair comparison of cells prepared in a single growth process, without the need to prepare multi-cultures in a sequential, thus time-dependent and potentially erroneously heterogenous, way.

## 5. Conclusions

In conclusion, we have developed the first, to the best of our knowledge, MLS offering a cost-effective, quantitative, and label-free method for simultaneous examination of multiple live cell cultures. Our innovative system, which integrates three lensless microscopes, enables time-lapse analysis of cell migration and provides quantitative measurements such as average cell speed in response to exogenous stimuli. By proposing a novel algorithmic approach for robust cell space-time segmentation and tracking, this easily scalable method can pinpoint the effect of stimulus (in our proof-of-concept case*:* chemical drug) on specific changes in migration activity. Leveraging the large FOV inherent to LDHM, our MLS allows for the observation of thousands of cells in vivo with single-cell precision, overcoming the limitations of traditional microscopy's restricted FOV. We believe this advancement will empower performing reliable live cell multi-culture comparisons and enhancing label-free investigations into cell cultures and the effects of biochemical or physical stimulants over large areas. Ultimately, this technology has potential to unlock new mechanistic insights through quantitative time-lapse observations of cell culture’s spatiotemporal optical signatures. For future studies, a standardized set of parameters could be proposed, to ensure methodological consistency, in close collaboration with biology experts.

## Supplemental information

Visualization 1 Time-lapse phase reconstruction of cell culture maged by camera 1. https://doi.org/10.6084/m9.figshare.27020032

Visualization 2 Time-lapse phase reconstruction of cell culture maged by camera 2. https://doi.org/10.6084/m9.figshare.27020041

Visualization 3 Time-lapse phase reconstruction of cell culture maged by camera 3. https://doi.org/10.6084/m9.figshare.27020053

## Figures and Tables

**Fig. 4.**
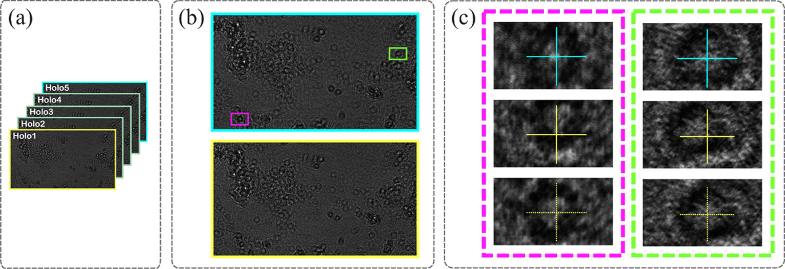
Rescaling hologram process: (a) set of five holograms with different defocus distances with first hologram named Holo1 (yellow frame) and last hologram Holo5 (blue frame), (b) enlarged Holo1 and Holo5 with marked two border areas, (c) enlarged areas depicted for Holo5 (top), Holo1 without rescaling (mid) and Holo1 after rescaling (down).

**Fig. 5.**
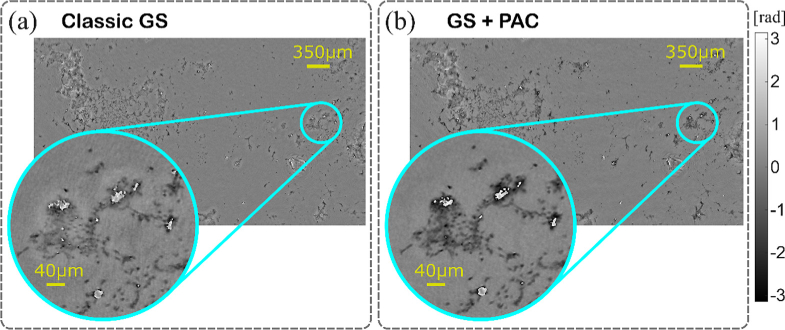
Comparison of reconstructions performed employing (a) classic GS and (b) new GS + PAC methods with exemplifying enlarged area, demonstrating signal improvement. Same colorbar was used in (a) and (b).

**Fig. 6.**
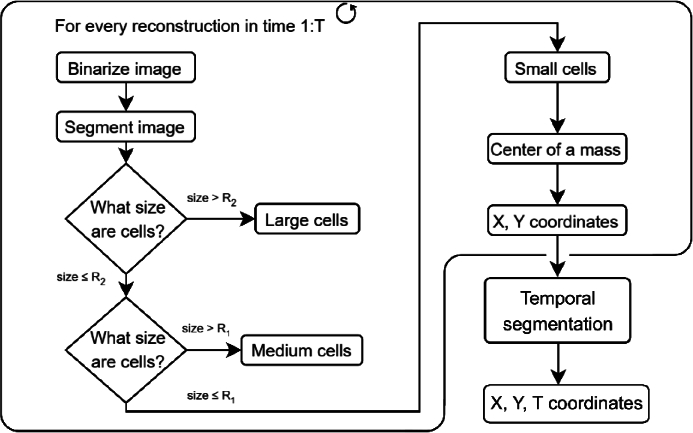
Diagram of labeling and segmenting process for every reconstruction.

## Data Availability

Data underlying the results presented in this paper are not publicly available at this time but may be obtained from the authors upon reasonable request.

## Appendix

We detail our method for computational reconstruction of holograms obtained using the lensless digital holography microscope (LDHM). To minimize twin-image artifacts, each reconstruction was generated from five recorded frames (holograms). These frames were captured within the same setup but with varying distances between the camera plane and the object plane. The first frame was taken at the closest possible camera position to the object, with subsequent frames captured at increasing distances, each spaced 1 mm apart. The acquisition time for a single frame was approximately 0.013 seconds, with camera movement time around 1 second, resulting in a total acquisition time of about 10 seconds (due to additional image saving time) for a set of 5 holograms. Given an average cell speed of 0.7 µm/min (see Fig. 3, blue line), cells required around 2 minutes to traverse a distance equal to one pixel. Thus, the acquisition time for all five frames was about 12 times faster than the time needed for cells to move by a single pixel, meaning that during the measurements required for a single reconstruction cells can be considered as static. At chosen reconstruction rate (1 per 5 minutes), cells can move up to approximately 2.5 pixels between successive reconstructions.

The optical magnification in LDHM can be calculated using the equation M = L/z, where L is the distance between the point source and the camera sensor, and z is the distance between the point source and the object. Here, the magnification of each collected hologram ranged from 1.11 to 1.15. Despite the seemingly small differences in magnification, its influence is significant due to the large field of view. The difference in the magnification between frames acquired at various sample-camera distances manifests in the holographic fringes displacement from the optical axis, proportional to both the magnification and distance from optical axis. To mitigate that effect, the holograms need to be rescaled accordingly to their magnification.

The hologram with the greatest magnification was the fifth one, marked with blue borders in Fig. 4 presented in the Appendix to enable appreciation of the novel algorithmic path employed in the MLS. Holograms 1-4 were scaled to match the hologram no. 5 (referred to as Holo5 in Fig. 1) to ensure accurate image features positions with pixel-wise precision. To appropriately resize holograms, we begin with numerical backpropagation of each hologram to the sample plane. Further, we automatically extract characteristic features from the reconstructed phases using the Speeded-Up Robust Features method [62]. Next, using the features identified in the reconstructions of currently processed hologram and the Holo5, we calculate 2-D geometric affine transformations [63]. These transformations are then applied to holograms 1-4 [64] ensuring correction of magnification and any potential lateral displacement in relation to Holo5.

On the left side of Fig. 4, a set of holograms used to reconstruct a single frame is shown. Cyan and yellow colours represent the fifth and first collected holograms, respectively, shown also in the central part of Fig. 4. Two regions (marked in the central part of Fig. 4 with pink and green) near the edges were selected to illustrate the scaling process. Boxes with dashed lines illustrate the same regions of interest (ROIs) at the camera plane. The first row represents ROIs from Holo5, with crosses marking the center of the features. The second row shows Holo1 with the same ROIs and the same crosses as in Holo5. The centers of the crosses in the second row indicate the need for scaling, as they do not mark the center of the features. The last row illustrates the ROIs from Holo1 after scaling. Crosses’ positions are consistent, and after scaling, the yellow dotted crosses (Holo1) align with the blue crosses (unscaled Holo5), indicating accurate feature alignment. The scaling process ensures compensation for differences in magnification, improving the accuracy of the final reconstructed image. The steps involved in this process are crucial for achieving high-quality reconstructions in LDHM.

After rescaling all collected holograms with abovementioned procedure, the complex optical field (*R*) in the object plane is retrieved with an iterative Gerchberg-Saxton (GS) algorithm with additional absorption constraint to improve algorithms’ convergence and signal-to-noise ratio.

The employed processing path is composed from nine steps described below:

(1) Begin the first iteration by assuming that optical field $(O_{1})$ in first hologram $(H_{1})$ plane is equal to the square root of this hologram: $O_{1}=\sqrt{H_{1}}$ .
(2) Propagate the $O_{1}$ to the sample plane (at $z_{1}$ ) with angular spectrum [38,39,65] method: $R=AS(O_{1},z_{1}).$
(3) Apply the absorption constraint to the *R* – amplitude values of *R* (|*R*|) that are larger than median values of |R| in first iteration are equalized to the mentioned median value. This constraint forces the algorithm to assume that the sample can only absorb the light and cannot produce the light. It is inspired by multi-wavelength complex field filtering algorithm [55], however newly modified and for the first time employed to multi-height holographic system.
(4) Backpropagate the *R* to the second hologram plane: $O_{2}=AS(R,-z_{2})$ .
(5) Replace the amplitude part of $O_{2}$ with the square root of the second hologram: $O_{2}=\frac{O_{2}}{|O_{2}|}\sqrt{H_{2}}$ .
(6) Propagate the $O_{2}$ to the sample plane: $R=AS(O_{2},z_{2})$ .
(7) Repeat steps (2) – (6) for each hologram.
(8) Repeat steps (2) – (7) for *N* iterations to improve results convergence (in our case N=5 , for larger *N* the reconstruction improvement was insignificant).
(9) After the last iteration, the obtained *R* is the reconstructed optical field at object plane

Contrary to the classical GS method without object absorption constraint (step (3) in above algorithm), the employed algorithm retrieves the cells phase information more efficiently, especially for low frequency phase information, Fig. 5. It should be noted that in both reconstructions, cells are displayed in negative phase values with respect to the background, due to the chosen illustrative convention for enhancing visual aspects.

For labeling (into individual cells, small and large clusters) and tracking cells, we used the algorithm illustrated in the diagram in Fig. 6. Firstly, each phase reconstruction was binarized with a manually adjusted threshold, consistent across all cell cultures, and then segmented (each separated binary cluster was treated as a separate object). After that, labeled objects were classified basing on their size: (1) objects larger than R2 (for mGRPs R2 was empirically set to 46 µm^2^) were marked as large cell clusters, (2) objects larger than R1 (23 µm^2^ for mGRPs) but smaller than R2 were marked as small cell clusters and (3) objects smaller than R1 were classified as individual (small) cells. Next, each individual object was spatially localized via calculation of its center of mass. After processing each reconstructed frame, the same individual objects were connected across subsequent frames to track their movement in time (temporal segmentation). This operation was done basing on the distance criterion (closest object across adjacent frames were marked as the same object).
